# Gut Microbiota Secondary Metabolites: Key Roles in GI Tract Cancers and Infectious Diseases

**DOI:** 10.3390/biomedicines13010100

**Published:** 2025-01-03

**Authors:** Eman K. E. Anwer, Muhammad Ajagbe, Moustafa Sherif, Abobaker S. Musaibah, Shuaib Mahmoud, Ali ElBanbi, Anwar Abdelnaser

**Affiliations:** 1 Biotechnology Graduate Program, School of Sciences and Engineering, The American University in Cairo, New Cairo 11835, Egypt; emananwer@aucegypt.edu (E.K.E.A.); d1stadeyemi@aucegypt.edu (M.A.); moustafa.sherif@aucegypt.edu (M.S.); 2 Department of Microbiology and Immunology, Faculty of Pharmacy, Modern University for Technology and Information, Cairo 4411601, Egypt; 3 Institute of Global Health and Human Ecology, School of Sciences and Engineering, The American University in Cairo, New Cairo 11835, Egypt; abobaker@aucegypt.edu (A.S.M.); mahmoudshuaib@aucegypt.edu (S.M.); 4 Biology Department, School of Sciences and Engineering, The American University in Cairo, New Cairo 11835, Egypt; alielbanbi@aucegypt.edu

**Keywords:** gut microbiome, secondary metabolites, cancer, infectious disease, gastro-intestinal tract, tumorigenesis, epigenetic regulation

## Abstract

The gut microbiota, a dynamic ecosystem of trillions of microorganisms, produces secondary metabolites that profoundly influence host health. Recent research has highlighted the significant role of these metabolites, particularly short-chain fatty acids, indoles, and bile acids, in modulating immune responses, impacting epigenetic mechanisms, and contributing to disease processes. In gastrointestinal (GI) cancers such as colorectal, liver, and gastric cancer, microbial metabolites can drive tumorigenesis by promoting inflammation, DNA damage, and immune evasion. Conversely, these same metabolites hold therapeutic promise, potentially enhancing responses to chemotherapy and immunotherapy and even directly suppressing tumor growth. In addition, gut microbial metabolites play crucial roles in infectious disease susceptibility and resilience, mediating immune pathways that impact pathogen resistance. By consolidating recent insights into the gut microbiota’s role in shaping disease and health, this review underscores the therapeutic potential of targeting microbiome-derived metabolites for treating GI cancers and infectious diseases and calls for further research into microbiome-based interventions.

## 1. Introduction

In recent years, the human gut microbiome has emerged as a central regulator of human health and disease, influencing processes from immune modulation [1,2,3] to epigenetic regulation [4,5,6]. Comprising an estimated 100 trillion microorganisms [7,8], the gut microbiome produces a wide array of secondary metabolites that extend beyond local gut health, influencing systemic immune responses, inflammatory pathways, and even the progression of complex diseases such as cancer [9,10].

The gut microbiota represents a highly dynamic and diverse microbial ecosystem, encompassing bacteria, archaea, fungi, and viruses that collectively play a pivotal role in maintaining host homeostasis. These microorganisms perform a range of essential functions, including nutrient metabolism, pathogen defense, and the modulation of immune responses. The microbiota’s composition is influenced by various factors, such as diet, age, antibiotics, and environmental exposures, and disruptions to its balance, termed dysbiosis, have been implicated in the onset and progression of numerous diseases, including cancer and infectious diseases [11,12]. Moreover, the gut microbiota serves as a biochemical factory, converting dietary components and endogenous substrates into bioactive metabolites that extend their effects far beyond the gastrointestinal tract [13]. These metabolites not only mediate local intestinal functions but also have systemic implications, influencing distant organs and disease pathways.

Among the most notable microbial metabolites are short-chain fatty acids (SCFAs), indoles, polyamines, and secondary bile acids, all of which have been shown to influence disease outcomes. For instance, Yao et al. [14] and Pant et al. [15] identified short-chain fatty acids (SCFAs) such as butyrate, acetate, and propionate as critical regulators of immune tolerance and inflammatory responses. Similarly, indole derivatives—products of bacterial tryptophan metabolism—have emerged as crucial mediators of immune homeostasis and cancer suppression. Scott et al. [16] demonstrated that indoles interact with the aryl hydrocarbon receptor (AhR) to modulate cytokine production and intestinal barrier function, positioning these metabolites as critical modulators of infection susceptibility and tumor progression. More recently, indole-based therapies have been shown to possibly enhance the efficacy of immune checkpoint inhibitors (ICIs), a breakthrough in cancer immunotherapy [17].

The ability of gut microbial metabolites to modulate epigenetic regulation has become an exciting frontier in the treatment of cancer and infectious diseases. The capacity of these metabolites to influence DNA methylation, histone modifications, and microRNA expression presents a novel therapeutic avenue for reprogramming disease-associated epigenetic alterations [18,19,20,21]. Butyrate, for example, has emerged as a potent histone deacetylase (HDAC) inhibitor, reshaping the expression of tumor suppressor genes and maintaining the epigenetic integrity of immune cells [22,23]. Recent work by Zhu and colleagues reinforced these findings, showing that butyrate not only modulates HDAC activity but also promotes cytotoxic T-cell function, enhancing the host’s ability to mount an immune response against tumor cells [24]. 

Epigenetics plays a pivotal role in the progression of cancer, acting as a crucial regulatory layer that enables mammalian cells to adapt their gene activity to environmental factors and influences gene expression without altering the underlying DNA sequence. Epigenetic modifications, including DNA methylation, histone modification, and the action of non-coding RNAs, significantly contribute to the dynamic process of tumorigenesis and cancer advancement [25,26]. While genetic mutations initiate tumorigenesis, the subsequent epigenetic alterations often facilitate the clonal evolution of cancer cells, enabling them to adapt to selective pressures and acquire malignant traits [27,28].

The immune-modulatory effects of microbial metabolites also extend to infectious diseases, which influence vital markers such as cytokines, C-reactive protein (CRP), and tumor necrosis factor-alpha (TNF-α). *Bifidobacterium longum* and *Lactobacillus rhamnosus* promote the production of anti-inflammatory cytokines such as interleukin-10 (IL-10), which mitigates systemic inflammation and enhances the host’s resistance to viral and bacterial infections [29,30]. This interplay between microbial metabolites and immune regulation forms the basis for their potential therapeutic use in controlling infectious diseases.

In the context of cancer, gut microbial metabolites have also been implicated in both tumor suppression and progression. The dual role of secondary bile acids, for instance, has been linked to colorectal cancer, where they can either promote tumorigenesis or suppress cancer growth depending on their concentration and the microbial context [31,32]. Furthermore, it was reported that polyamines, another class of microbial metabolites, regulate oncogenic microRNAs such as miR-21 and miR-155, both of which are involved in cancer cell proliferation and metastasis [33]. This microRNA regulation highlights the intricate role of microbial metabolites in modulating gene expression related to cancer development.

Taken together, it illuminates a previously underappreciated axis of disease regulation—one in which the gut microbiome exerts profound control over epigenetic and immune pathways, influencing both infectious disease susceptibility and cancer progression. This review aims to synthesize the emerging evidence surrounding the therapeutic potential of gut microbiome-derived secondary metabolites, with a focus on their role in epigenetic regulation. By examining the intricate interplay between the microbiome, epigenetic mechanisms, and disease markers, we seek to highlight novel opportunities for therapeutic intervention in the management of both chronic infections and cancer.

## 2. The Roles of Gut Microbiota Metabolites in the Development of GI Tract Cancers

The gut microbiota potentially metabolizes our diets, producing numerous bioactive secondary metabolites that accomplish interactions between the host cell and the microbe [34]. Recent research interest in gut microbiota metabolites sheds light on their role in tumor development or prevention [35]. We discuss the gut microbiota metabolites that impact colorectal, liver, esophageal, gastric, and pancreatic cancers.

### 2.1. Colorectal Carcinoma (CRC)

**Figure 1:**
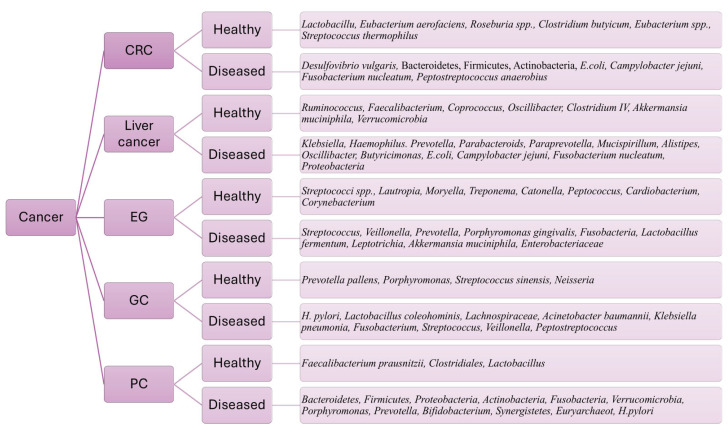
Gut microbiota alterations in GIT cancers. This figure illustrates the differences in gut microbiota composition in cases of healthy individuals and patients with GIT cancers [36,37,38,39,40,41,43,44,45,46,47,48,49,50,51,52,53,54,55,56]. Created with Google Slides.

Moore and Moore [36], Swidsinski et al. [37], Wang et al. [38], and O’Keefe et al. [39] reported in their studies that sulfur-reducing/hydrogen sulfide-producing bacteria, such as *Desulfovibrio vulgaris*, and secondary bile acid (BA)-producing bacteria are more prominent with a high risk of colorectal cancer (Figure 1) [40]. Hydrogen sulfide produced by sulfur-reducing gut microbiota was found to have cytotoxic and genotoxic activities, as it could damage DNA, stimulate genes involved in the nuclear factor kappa B (NF-κB) pathway, and inhibit anti-inflammatory responses (Table 1), thereby stimulating CRC cell proliferation (Table 2) [41,42].

BAs are the primary metabolites of cholesterol in the liver, and these BAs are then metabolized into secondary BAs by specific gut microbiota (Bacteroidetes, Firmicutes, and Actinobacteria phyla) (Figure 1) in the intestine [35,57]. Deoxycholic acid (DCA) and lithocholic acid (LCA) are secondary BAs that decrease the activity of the farnesoid X receptor (FXR); it is a nuclear receptor that has a tumor-suppressive effect and is involved in the development of CRC [58,78]. This FXR activity reduction stimulates the Wnt/β-catenin pathway, leading to DNA damage, apoptosis prevention, and consequently cancer development [35,58]. Secondary BAs also upregulate interleukin-8 (IL-8) (Table 1), which is an oncogene, inducing ERK1/2 pathways, and are also able to stimulate the MAPK pathway, resulting in CRC development (Table 1) [41]. Mohseni et al. also reported that lactate, a metabolite produced through gut microbiota metabolism, is an oncometabolite that facilitates the transport of oxygen and other nutrients to the CRC cells and increases the PH value of the tumor microenvironment (TME) (Table 1). Therefore, lactate induces CRC cell proliferation and metastasis [40]. Contrary to this study, Cheng et al. reported that lactate-producing gut microbiota such as *Streptococcus thermophilus* (Figure 1) may have protective activity against CRC (Table 1) [42,43].

*Enterotoxigenic Bacteroides fragilis* produces a carcinogenic toxin that stimulates cell proliferation (Table 1) by inducing the β-catenin/Wnt signaling pathway, induces the release of proinflammatory factors, and causes DNA damage (Table 1) [42,59,60].

Parallelly, there has also been mention of genotoxins produced by various bacteria in the gut that do play a role in CRC progression. One example is the *E. coli* genotoxin known as colibactin. Specifically, the *E. coli* strain from phylogenetic group B2 contains a genomic island called “polyketide synthase (pks)”, which is responsible for colibactin production. Colibactin causes gene mutations, DNA damage, and chromosomal instability in the colonocytes, inducing colon tumorigenesis. Colibactin improves the proliferation efficacy of tumor cells (Table 1) [43,60,61,62]. Another study vaguely describes a cytolethal toxin produced by *Campylobacter jejuni*, causing DNA double strands to break and stimulating colon tumorigenesis (Table 1) [43], in addition to adhesin A toxin produced by *Fusobacterium nucleatum* (Table 1), which is involved in the development of CRC (Table 1) [44]. Due to their pathogenic role, gut metabolites may serve as biomarkers for CRC diagnosis and follow-up on disease status during or after treatment (Figure 1).

### 2.2. Liver Cancer

Chronic liver diseases (CLDs) are mainly attributed to hepatocyte inflammation, which leads, over time, to a severely injured liver, fibrosis, cirrhosis, and then hepatocellular carcinoma (HCC). The gut microbiota and their metabolites play a role in liver inflammation, fibrosis, and HCC via the “gut–liver axis” [66,79,80]. Ren et al. and colleagues reported in their study that butyrate-producing bacteria such as *Ruminococcus*, *Faecalibacterium*, *Coprococcus*, *Oscillibacter*, and *Clostridium IV*, in addition to *Akkermansia muciniphila* and *Verrucomicrobia*, were reduced in early HCC patients, while lipopolysaccharide (LPS) producers such as *Klebsiella* and *Haemophilus* (Table 1) were more abundant in HCC patients than in controls (Figure 1) [45,46]. Butyrate plays a pivotal role in promoting host cell differentiation, inflammation, apoptosis, and intestinal mucosal integrity; thus, butyrate depletion contributes to HCC development (Table 1) [63].

Conversely, increased LPS levels boost HCC progression by stimulating the production of proinflammatory cytokines (Table 1) [66,67]. These LPSs potentially disrupt the epithelial tight junctions (TJs) of the intestine, increasing the leakage of endotoxins produced by gut microbiota. They disrupt the TJs through the induction of the “toll-like receptor (TLR) 4–myeloid differentiation primary response 88 (MyD88)” pathway, stimulating the NF-kB pathway. The NF-kB pathway then induces Kupffer cells to produce proinflammatory cytokines, causing severe hepatitis followed by carcinogenesis [68]. Similarly, Gupta et al. documented that LPS-producing bacteria participate in HCC pathogenesis (Table 1), including *Prevotella*, *Parabacteroids*, *Paraprevotella*, *Mucispirillum*, *Alistipes*, *Oscillibacter*, and *Butyricimonas* (Figure 1) [47]. *E. coli* was increased considerably in HCC patients [48]. A tumorigenic gut microbiota causes DNA damage due to genotoxin production; for instance, colibactin produced by *E. coli* and “cytolethal distending toxin” synthesized by *proteobacteria* and *Campylobacter jejuni* stimulated double-stranded DNA breakage (Table 1). *F. nucleatum*, *Bilophila wadsworthia*, and *Desulfovibrio desulfurican* (Figure 1) produce reactive oxygen species, stimulating DNA oxidative stress [49].

### 2.3. Esophageal Cancer (EC)

Studies have revealed that the composition of gut bacteria (microbiota) is different in people with esophageal cancer compared to those without. Patients with esophageal squamous-cell carcinoma (ESCC) and esophageal adenocarcinoma (EAC) have lower overall bacterial diversity than healthy individuals. Certain types of bacteria are more common in people with these cancers, whereas others are less common. For example, *Streptococcus*, *Veillonella*, *Prevotella*, *Porphyromonas gingivalis* [50,81,82,83], and *Fusobacteria* are more abundant in ESCC (Figure 1) [50]. Chiang et al. [51] reported in their review that *P. gingivalis* stimulated ESCC cell growth and invasiveness via NF-KB pathway activation, which was also shown in Meng et al.’s study in 2019 [84]. Similarly, Chiang et al. discussed the effect of *F. nucleatum* (Figure 1) on ESCC cell proliferation and migration [51]. Another study indicated that *P. gingivalis* and *F. nucleatum* LPSs stimulate the NF-KB pathway by triggering the TLR-4/MyD88 cascade. NF-kB activation induces proinflammatory cytokine production, resulting in severe inflammation followed by carcinogenesis (Table 1) [69,70]. On the other hand, Nomoto et al. indicated in their study that there were no changes in TLR-4 expression associated with *F. nucleatum*, despite their conformity with the participation of *F. nucleatum*, mainly in NF-kB activation and the NOD-like signaling pathway [85].

### 2.4. Gastric Cancer (GC)

Gastric cancer is an inflammation-induced cancer, and infection with *Helicobacter pylori* promotes the immune system and inflammation; hence, *H. pylori* is a risk factor (Figure 1) for GC and could be targeted to protect against GC [52]. It was reported that *H. pylori* produces toxic proteins such as vacuolating cytotoxin A (VacA), cytotoxic-associated gene A (CagA), and “outer membrane proteins”. The phosphorylated CagA stimulates proteins that are responsible for stomach cell morphology, causing cell elongation and scattering. Other CagA proteins stimulate interleukin release via activating the transcription factor NF-B, in addition to their mutagenic effect (Table 1) [72,73]. In contrast, people without *H. pylori* have more *Firmicutes*, *Bacteroidetes*, and *Actinobacteria* (Figure 1) [53]. Changes in gut bacteria (microbial dysbiosis) are linked to stomach cancer [53]. Studies have found that people with stomach cancer have a wider variety of gut bacteria, with more *Lactobacillus coleohominis*, *Lachnospiraceae*, *Acinetobacter baumannii*, and *Klebsiella pneumoniae* [54]. Liu et al. discussed several bacterial genera associated with GC progression in several datasets: *Fusobacterium*, *Streptococcus*, *Veillonella*, and *Peptostreptococcus* (Figure 1) [55]. Interestingly, these gut microbiotas were reported to induce GC through lactic acid production and are known as lactic acid bacteria (LAB) [86]. Lactate production induces tumor cell progression and fuels the GC cells. In addition to lactate, they could convert nitrate into nitrite, producing N-nitroso metabolites in massive amounts, which stimulate the epithelial cells to induce mutagenesis, angiogenesis, and proto-oncogene expression. In an in vitro study, this group of bacteria was found to generate “reactive oxygen species (ROS)”, consequently inducing DNA damage. Furthermore, LAB promotes NANOG, a “multipotency marker” that participates in the transformation of fibroblasts into multipotent cells [86,87].

### 2.5. Pancreatic Cancer (PC)

It was shown that pancreatic cells can be affected by gut microbiota. Pancreatic cells produce an antimicrobial molecule called “cathelicidin-related antimicrobial peptide” (CRAMP) against pathogenic/non-required gut microbiota [88]. Butyrate and acetate are produced by gut microbiota; butyrate stimulates pancreatic cells to produce CRAMP [89], whereas acetate induces insulin release [90]. Gut microbiota dysbiosis is linked to enhanced inflammation conditions, proliferation, angiogenesis, invasion, metastasis, and immunity modulation [91].

*H. pylori* infection has been linked to an increased risk of pancreatic cancer (PDAC), potentially by promoting cell proliferation [56,92]. *H. pylori* potentially produces many oncometabolites, such as LPS and ammonia, in addition to inducing the production of inflammatory cytokines. *H. pylori*-derived LPSs can promote KRAS mutations, leading to the development of PC [54]. Ren et al. specifically observed an increase in harmful LPS-producing bacteria, where increased LPS levels potentially activate the NF-kB pathway, and a decrease in beneficial probiotics and butyrate-producing bacteria in PDAC patients (Table 1) [56,71].

## 3. Impact of Gut Microbiota Metabolites on Cancer Treatments

### 3.1. Chemotherapy

Drug resistance significantly affects treatment efficacy and patient survival, leading to varied drug responses. The gut microbiome plays a crucial role in processing chemotherapy drugs, influencing their toxicity and effectiveness via their products. Dysbiosis, often caused by antibiotic use, can hinder the function of chemotherapy and may even contribute to cancer progression [93,94]. Research has identified three ways by which the gut microbiome influences chemotherapy: (1) enhancing drug effectiveness, (2) neutralizing cancer properties, and (3) managing drug toxicity. These findings underscore the importance of considering the gut microbiome in personalized cancer treatment plans, with consistent evidence linking gut bacteria to the effectiveness of chemotherapies and advanced immunotherapies [94,95].

#### 3.1.1. Irinotecan

**Figure 2:**
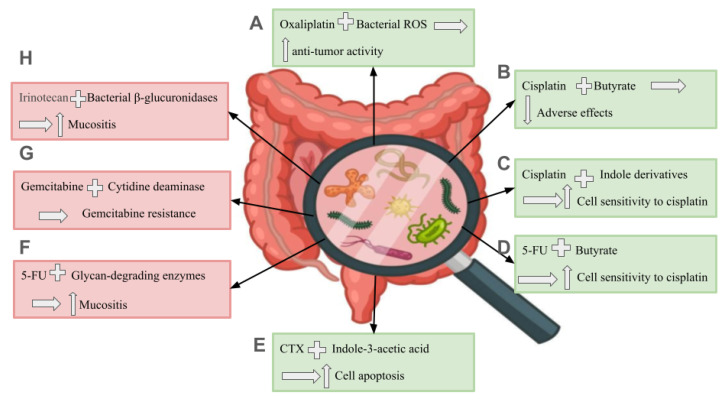
Gut microbiota metabolites and their effect on different chemotherapeutic agents. This figure shows the positive (green boxes) and negative (red boxes) impact of the secondary products of the gut microbiota on anticancer treatments. (**A**) The enhancement potentiality of the bacterial-produced ROS on oxaliplatin’s anti-tumor activity. (**B**,**C**) The benefits of butyrate and indole derivatives (gut microbiota metabolites) on diminishing cisplatin-induced adverse effects and increasing the tumor cells’ sensitivity to the drug, respectively. (**D**) The increased tumor cell sensitivity to 5-FU due to the high level of butyrate, and (**E**) the enhanced cell apoptotic activity of CTX in the presence of indole-3-acetic acid. Meanwhile, (**F**) indicates 5-FU-induced mucositis with increased glycan-degrading enzyme abundance. (**G**) The metabolic activity of the bacterial cytidine deaminase on Gemcitabine-inducing tumor cell resistance. Finally, (**H**) represents the effect of the bacterial β-glucuronidases on Irinotecan-induced mucositis. ROS (reactive oxygen species), 5-FU (5-Fluorouracil), CTX (Cyclophosphamide). Created with Google Slides.

Irinotecan is an anticancer drug of the “DNA topoisomerase I inhibitor” class. It is used to treat solid tumors (such as colorectal, ovarian, pancreatic, and lung cancers). Irinotecan is an active drug that is activated to produce the SN-38 active metabolite by hydrolysis, specifically by the carboxylesterase enzyme, and deactivated by the ability of UDP-glucuronyltransferases to produce its inactive SN-38G form [96]. SN-38G is reactivated because of the effect of the enteric bacterial β-glucuronidases inside the gut, resulting in SN-38- or irinotecan-induced mucositis (Figure 2). Administration of ciprofloxacin and low doses of amoxapine has modulated the impact of the bacterial β-glucuronidases [97,98,99,100]. 

#### 3.1.2. Gemcitabine

Gemcitabine is an antimetabolite antineoplastic drug mainly used to treat solid tumors, such as non-small-cell lung, pancreatic, breast, and ovarian cancers. It is also used off-label in bladder cancer. Gemcitabine is a pyrimidine analogue that inhibits DNA synthesis, leading to cell apoptosis [101]. The cytidine deaminase (CDD) enzyme is responsible for its metabolism into the inactive difluoro-deoxy-uridine compound. Geller et al. and Choy et al. reported in their studies on colon cancer and pancreatic ductal adenocarcinoma that *Gammaproteobacteria*, such as *E. coli*, have the ability to produce the bacterial long-isoform cytidine deaminase enzyme, increasing the gemcitabine biodegradation that results in gemcitabine resistance (Figure 2) [99,102,103].

#### 3.1.3. Oxaliplatin

Oxaliplatin is a platinum-based antineoplastic drug that is mainly used in metastatic CRC and after stage III CRC resection surgeries. It has dose-limited toxicity on the nervous system, causing mechanically sensitive severe pain known as “mechanical hyperalgesia” [104]. It was found that the gut microbiota strengthens both its antitumor and neurotoxicity effects. This synergism comes from the impact of both the gut microbiota and oxaliplatin on the immune system and the inflammatory response [98,105,106,107].

Bacterial ROS enhance the anti-tumor efficacy of oxaliplatin (Figure 2) [108,109]. In contrast, Yu et al. observed that *F. nucleatum* potentially promotes the expression of LC3-II “autophagic reflux” and the synthesis of autophagosomes, as well as the expression of autophagy-related proteins with undetected mechanisms and a critical metabolite, resulting in CRC cells’ resistance to oxaliplatin [110].

#### 3.1.4. Cisplatin

Cisplatin is a platinum-based antineoplastic drug and an alkylating agent. It is used to control and treat solid tumors and hematologic cancers. It has serious toxic effects, such as ototoxicity, neurotoxicity, myelosuppression, and gastrointestinal toxicity [111]. The gut microbiota can provide a protective effect against cisplatin-induced adverse effects. The Lachnospiraceae family are butyrate producers responsible for the anti-inflammatory and antimicrobial effects against pathogenic bacteria [99,112,113,114]. Hence, they alleviate cisplatin-induced intestinal epithelium damage (Figure 2) [115,116]. In addition, Ling Hui reported that lactobacillus spp. have antibacterial, antioxidant, and anti-inflammatory effects on the intestine, reducing cisplatin-induced mucositis [116]. In addition to its impact on cisplatin-induced mucositis, lactobacillus species also reduce the weight loss and cardiac dysfunction associated with cisplatin administration (Figure 2) [99,117]. Furthermore, Chambers et al. revealed that gut microbiota produced indole-3-propionic acid and indoxyl sulfate metabolites, potentially sensitizing epithelial ovarian cancer cells to cisplatin treatment (Figure 2) [118].

#### 3.1.5. 5-Fluorouracil (5-FU)

Fluorouracil is a systematic chemotherapeutic drug used in breast, gastric, colorectal, and pancreatic adenocarcinomas. It also has off-label uses with biliary tract carcinoma, esophageal cancer, anal carcinoma, and cervical cancer. In addition, it could be topically prescribed in different dermatological cases. It causes several adverse effects when systematically administered; severe mucositis is one of the frequently occurring adverse effects with 5-FU treatment strategies that may cause drug discontinuation or dose reduction [119]. Hamouda et al. reported that Gram-negative gut microbiota species participate in 5-FU-induced secondary inflammation post-intestinal crypt apoptosis one day after drug administration. She observed that diminished *firmicutes* (Gram-positive) and increased *Bacteroidetes* and *Verrucomicrobia* (mainly Gram-negative) bacteria promote 5-FU-induced mucositis [120]. Similarly, Yuan et al. found that an increased abundance of *Enterobacteriaceae*, *Lachnospiriaceae*, and *Bacteroidaceae* is involved in the pathological mechanism of 5-FU-induced intestinal mucositis [121].

Members of the *Bacteroidaceae* family can increase the incidence of colitis in animals by producing glycan-degrading enzymes (Figure 2) [121,122]. *Lactobacillus plantarum* produced metabolites that were found to enhance 5-FU anti-tumor activity, as these metabolites augment butyrate transporter expression. Butyrate mainly sensitizes tumor cells to 5-FU by inhibiting the glucose metabolism pathway (Figure 2) [109]. On the other hand, *F. nucleatum* was observed to induce 5-FU resistance in CRC cells by inducing autophagic flux and its associated proteins [110,123].

#### 3.1.6. Cyclophosphamide (CTX)

Cyclophosphamide is an alkylating agent that acts as a chemotherapeutic and immunosuppressant drug. CTX is mainly prescribed in the advanced stages of malignant lymphoma, multiple myeloma, breast cancer, ovarian adenocarcinoma, retinoblastoma, disseminated neuroblastomas, and sarcoma. It is also used for autoimmune diseases such as multiple sclerosis and pretransplant surgeries [124]. CTX causes DNA alkylation and inhibits protein synthesis due to DNA and RNA crosslinking. Besides its antitumor effects, CTX can modulate the immune system by reducing interferon-gamma (INF-Υ) and interleukin-12 (IL-12) secretion and stimulating the release of T-helper 2 (Th2) cytokines such as IL-4 and IL-10 in the peripheral blood and cerebral spinal fluid (CSF) [124]. It has been documented that *Enterococcus hirae* translocation in the lymph nodes and *Barnesiella intestinihominis* localization in the colon promote CTX-induced immunogenic cancerous cell death through T helper 17 (Th17) and T helper 1 (Th1) cell regulation. Moreover, Th1 responses related to *E. hirae* and *B. intestinihominis* were found to be associated with survivors of lung and ovarian cancer who were treated with chemo-immunotherapy agents such as CTX [100,125,126]. These two microorganisms were found to convert tryptophan to indole-3-acetic acid (IAA) (Figure 2). IAA is then oxidized by myeloperoxidase, producing ROS that modulate chemotherapy-induced cell apoptosis (Figure 2) in addition to reducing autophagy accumulation [127,128]. Altogether, *E. hirae* and *B. intestinihominis* enhance CTX anti-tumor outcomes.

### 3.2. Immunotherapy

It was discovered that the gut microbiota has the ability to induce and enhance anti-tumor immune therapy responses by modulating the host’s immune system (CD8+ T, Th 1, and myeloid cells associated with tumors) [129].

#### 3.2.1. Programmed Cell Death Protein-1 Receptor (PD-1) Inhibitors

PD-1 is one of the T cell checkpoint proteins; it acts as an “off switch” protein. When it binds to its ligand PD-L1 protein, it may be expressed in cases of normal and cancerous cells and prevents immune cells (T cells) from attacking other cells. Some cancerous cells have vast amounts of PD-L1 that hide the cancer cells from immunity. PD-1 inhibitors such as Pembrolizumab, Cemiplimab, and Nivolumab [130] stimulate the suppressed immune system to activate anti-tumor immune cells [131]. Human and animal model studies reported that responses to treatment with PD-1 inhibitors are significantly associated with a high abundance of *Akkermansia muciniphilia*, the *Ruminococcaceae* family, *Bifidobacterium longum*, *Collinsella aerofaciens*, and *Enterococcus faecium.* They also revealed that fecal microbial transplantation from PD-1 inhibitor responder patients to germ-free animal models restores the drug’s anti-tumor efficacy [98,132,133,134,135,136,137].

For instance, *Bifidobacterium* was reported to produce hippurate metabolites after a high-salt diet (HSD), enhancing PD-1 inhibitor immunotherapy’s anti-tumor efficacy [138]. As previously discussed, *F. nucleatum* LPSs activate the NF-κB pathway [69,70]. Gao et al. observed that NF-κB activation due to *F. nucleatum* promotes expression of the PD-L1 protein, thus enhancing PD-1 inhibitor immunotherapy against colorectal tumor cells [139]. 

#### 3.2.2. Cytotoxic T-Lymphocyte-Associated Protein-4 (CTLA-4) Inhibitor

CTLA-4 is a protein found on the surface of T cells. In typical cases, CTLA-4 maintains immune function. While it binds to another protein called B7, T cell efficacy in fighting invaders and cancerous cells is controversial. When the CTLA-4 inhibitor drug (ipilimumab or tremelimumab) is administered, it hinders the binding of CTLA-4 and B7, freeing the T cells to attack tumor cells [99]. 

Vétizou et al. reported that the gut microbiota (*Bacteroides thetaiotaomicrom*) has the ability to boost the anti-tumor efficacy of CTLA-4 inhibitors by inducing Th 1 cell immune responses [136,137,140]. Moreover, the previously mentioned species can reduce CTLA-4 inhibitor-induced immune-related colitis, specifically the Bacteroidetes phylum [99,137,140,141]. Additionally, *Bacteroides fragilis* capsular polysaccharides can stimulate Th 1 cells, enhancing CTLA-4 inhibitor anti-tumor activity [140].

#### 3.2.3. Toll-like Receptor Agonists (TLR)

CpG-oligodeoxynucleotide (CpG-ODN) is a toll-like receptor 9 (TLR9) agonist. It is a synthetic, short, and single-stranded nucleic acid involving unmethylated cytosine–guanine dinucleotides known as “CpG motifs” developed from bacterial DNA. It acts as an immunoadjuvant through TLR9 stimulation, producing pro-inflammatory cytokines such as IL-2, IL-12, or INFΥ, and then INFΥ activates the Th1 cells [142,143]. Iida et al. stated that *Alistipes shahii* is positively associated with CpG ODN antitumor efficacy, as *A. shahii* is a tumor necrosis factor (TNF) inducer [100,144].

## 4. The Potential of Gut Microbiota Metabolites in GI Tract Cancer Therapeutics

In this section, we discuss three important classes of secondary metabolites and how some of their representatives can act as anticancer agents. Epigenetic modifications, such as DNA methylation, histone modifications, and non-coding RNA regulation, play significant roles in regulating gene expression. Aberrant epigenetic changes can lead to the silencing of tumor suppressor genes or the activation of oncogenes, contributing to cancer development and progression [145].

### 4.1. Short-Chain Fatty Acids (SCFAs)

#### 4.1.1. Butyrate

Butyrate, or butyric acid, is a well-defined SCFA that has been studied for its multiple beneficial roles and potential as a therapeutic [64,146,147,148,149]. One study experiment with butyrate supplements and demonstrates their multiple effects on cancer-related pathways. Specifically, through RNA-seq analysis, the study describes how butyrate supplementation upregulates calcium signaling pathways and promotes apoptogenic activities. By performing a knockdown of the ACADS gene, the gene responsible for SCFA metabolism, and supplementing with sodium butyrate (NaBu), researchers were able to describe dose-dependent inhibition of HCC cell growth (Table 1) [64]. The research also describes the effects of the two other well-known SCFAs on the same HCC cell lines (more below).

Research has demonstrated that the gut microbiota can modulate epigenetic mechanisms influencing cancer progression, such as DNA methylation and histone modification. Specific microbial metabolites, such as SCFAs, are produced through the fermentation of dietary fibers and can affect cellular energy metabolism and intestinal health and indirectly regulate epigenetic mechanisms involved in cancer development, such as DNA methylation, histone modifications, and non-coding RNAs [150]. SCFAs have been shown to exert epigenetic effects by modifying histone acetylation; for instance, acetate and propionate deactivate HDAC2 and 3 enzymes, while butyrate inhibits the activities of HDAC1 and 2, which affect gene transcription. They can also change DNA methylation patterns, altering gene expression [151,152,153,154]. The inhibition of HDACs can lead to the activation of tumor suppressor genes and the repression of oncogenes, thus reducing the risk of tumorigenesis. For example, butyrate has been associated with promoting apoptosis and inhibiting cancer cell proliferation [155,156].

Similarly, other researchers highlighted a direct link between NaBu and autophagy. Through inducing the production of ROSs, NaBu can induce autophagy in hepatoma cells [156]. The study explains how butyrate acts as a modulating agent of the Akt/mTOR pathway, which, in parallel, plays a significant role in hepatoma cell autophagy [156]. Another study discusses how NaBu can also enhance the efficacy of the chemotherapeutic agent oxaliplatin, which is mainly used to treat colorectal cancer (Table 1). This describes how NaBu, accompanied with oxaliplatin, can better inhibit colorectal cancer cell growth and proliferation [65]. 

Multiple other studies explore the role of butyrate as a backbone to many anticancer therapeutic agents. One such compound is tributyrin, which has been shown to exhibit a more potent (2.5–3.0-fold) effect with regards to cancer cell inhibition [157]. The study also explains how tributyrin has promoted apoptotic activity in prostate cancer cell lines [157]. Tributyrin was also described as exhibiting histone deacetylase inhibitor (HDACi) activity. A study concluded that tributyrin could induce apoptosis in primary and recurrent polymorphonuclear leukocytes (pPNLs and rPNLs), both of which play a major role in hepatocarcinoma [158]. 

Butyroyloxymethyl diethylphosphate (AN-7) and pivaloyloxymethyl butyrate, or AN-9, are other butyrate-based prodrugs that can also be classified as HDAC inhibitors. In a study using prostate carcinoma cells, both AN-7 and AN-9 were proven to have massive effects on cancer cells. In a dose-dependent manner, both compounds could induce apoptosis in human umbilical vein endothelial cells. Other than reducing the viability of the aforementioned cells, butyrate-based compounds also played a significant role in inhibiting the migration of cancerous cells [159]. 

#### 4.1.2. Acetate and Propionate

Just like butyrate, acetate, as a stand-alone molecule, has been studied extensively for a variety of reasons, including but not limited to industrial uses, biotechnological applications, and bioremediation experiments [160,161,162,163]. Regarding research on its potential as an anticancer therapeutic, the same cannot be said about either molecule. Despite some studies in the literature discussing acetate and propionate as backbones to some anticancer agents, there is still a lot to explore about the effects of each of these molecules on different types of cancer. 

A comprehensive study by Che and colleagues examines the anticancer potential of the three major short-chain fatty acids (SCFAs)—butyrate, acetate, and propionate—in hepatocellular carcinoma (HCC) [64]. Although butyrate is the primary focus, the researchers included sodium acetate and sodium propionate (NaAc/NaPr) as comparative agents. The findings demonstrate that all three SCFAs exert a dose-dependent inhibitory effect on HCC across two distinct cell lines, underscoring their potential as anticancer agents. NaBu displayed significantly more potent inhibition of HCC cell proliferation compared to NaAc/NaPr, with lower concentrations needed to achieve comparable effects. This study emphasizes the superior efficacy of NaBu in inhibiting HCC growth and highlights concentration-dependent variations in SCFA effectiveness.

Although research on acetate and propionate as anticancer agents remains limited, there is substantial interest in acetate derived from marine and plant microorganisms as a secondary metabolite with potential therapeutic applications [164,165,166,167,168]. Given that the focus of this review is confined to secondary metabolites produced by human microbiota, a detailed analysis of studies on acetate from non-human sources lies outside of our scope.

#### 4.1.3. Indirect Links Between Tertiary Compounds and SCFA Production and Activity

One study by Bamigade et al. discussed the relationship between extracted polysaccharides from date pumice and certain gut microbiota members’ production of SCFAs. Specifically, the polysaccharides, extracted via a microwave-assisted deep eutectic solvent, promoted the growth of *Gemmiger formicilis*, Blautia species, *Collinsella aerofaciens*, and *Bifidobacterium longum*, all of which are producers of SCFAs [169]. Another study demonstrates the critical role SCFAs play as anticancer agents by studying the effect of echinacoside (ECH) on SCFA-producing bacteria. The research concluded that ECH promotes the activity of SCFA-producing bacteria without compromising the total bacterial load [170]. The study particularly describes the dose-dependent growth of butyrate-producing *Faecalibacterium prausnitzii* and links the overall production of ECH-induced SCFA production to the inhibition of liver metastasis through the suppression of PI3K/AKT signaling [169]. From here, it is clear that there exists a variety of studies indirectly describing the anticancer properties of SCFAs by exploring the effects of tertiary compounds on the bacteria that produce them and observing the role they play against cancerous cells [169,170].

Furthermore, the integration of dietary interventions aimed at optimizing gut microbiota composition may serve as a complementary approach to conventional cancer treatments. Diets rich in fiber and polyphenols can promote the growth of beneficial microbiota, leading to increased production of SCFAs and subsequent epigenetic modifications that could reduce cancer progression [147]. This dietary modulation highlights the potential of a holistic approach to cancer prevention and treatment, emphasizing the need for further research in this area.

The therapeutic potential of manipulating the gut microbiota to influence cancer progression is gaining traction. Fecal microbiota transplantation (FMT) and probiotic supplementation are being explored as strategies to restore a healthy microbiome and potentially reverse epigenetic changes associated with cancer. Certain microbial species have been found to enhance the effectiveness of immune checkpoint inhibitors, leading to improved clinical outcomes in cancer patients [171].

### 4.2. Bacteriocins

Bacteriocins are a class of secondary metabolites produced by bacteria that have a role similar to antibiotics, except being tailored for a narrower spectrum of bacteria mostly closely related to the producing strain [172,173]. They can be better described as ribosomally synthesized antimicrobial peptides [173]. A vast majority of the existing literature explores bacteriocins’ relationship to food applications, antibiotic effects, and possible therapeutic applications for gut dysbiosis [173,174,175,176,177]. Here, we focused on two well-studied bacteriocins (nisin and pediocin) and their potential as anticancer therapeutic agents.

#### 4.2.1. Nisin

Thanjavur et al. (2022) discussed nisin’s antimicrobial and apoptogenic properties through its application to *E. coli* and multiple non-cancerous and cancerous animal cell lines [74]. The researcher observed high caspase-3 protein (a protein playing a vital role in the modulation of apoptosis) levels in healthy and cancerous cell lines following the application of nisin. Magnificently, the results show a synergetic relationship whereby the levels of caspase-3 increased significantly following the addition of nisin in cancerous cells but were significantly lower in healthy cell lines exposed to the same treatment. The results point towards the anticancer abilities of nisin, and it can be used as a candidate for anticancer therapeutics (Table 1) [74]. Another study describes the new role of the known apoptotic mediator CHAC1 as a promoter for cell apoptosis under nisin treatment [178]. Researchers used apoptosis, cell cycle, and in vivo toxicity assays to conclude that nisin decreases head and neck squamous-cell carcinoma (HNSCC) tumorigenesis in vivo and in vitro [178]. Yet another study confirms these findings, explaining nisin’s different roles in reducing tumorigenesis in HNSCC (Table 1) and inducing apoptosis in human umbilical vein endothelial cells (HUVECs) [75]. By diving into the effect of this bacteriocin on specific transcription factors, some studies demonstrate the dose-dependent manner in which Nisin could act as a therapeutic agent. Concerning liver cancer (HCC), researchers, through multiple assays and docking analyses, were able to identify that nisin downregulates the TWIST1 transcription factor, a known driver of epithelial-to-mesenchymal transition (EMT) and cancer progression [179]. From here, it becomes clear that nisin has huge potential as a therapeutic agent due to its ability to target head, neck, liver, and even colon cancers [74,75,178,179].

#### 4.2.2. Pediocin

Classified as a class 2 bacteriocin, pediocin is a less studied yet promising candidate for anticancer treatment [180]. A study examined the effect of pediocin K2a2-3 on lung and colon cancer cell lines and found that the peptide has an inhibitory effect on cellular progression (Table 1) [76]. Similarly, another study conducted an MTT assay with pediocin only on human cervical carcinoma (CC) and human colon adenocarcinoma (HCA) cell lines. Furthermore, the results supported pediocin’s anticancer abilities (Table 1). Specifically, pediocin showed high levels of cytotoxicity in both cell lines, leading the researchers to credit the results to the peptide’s hydrophobicity [77]. Pediocin CP2, another variant of the bacteriocin, was also studied for similar characteristics [181]. In this comparative study, native pediocin was tested against pediocin made through recombination using an MTT assay conducted on four different cell lines: CC, HCA, mammary gland adenocarcinoma, and spleen lymphoblasts [181]. Here, researchers concluded that the recombinant version of pediocin had a significantly higher level of cytotoxicity for all tested cell lines [181]. The work done by Kumar et al. is just one example of the many therapeutic opportunities made possible by class 2 bacteriocins in general and pediocin specifically. 

Despite limited studies on the potential of pediocin as an anticancer agent, there is rising interest in discovering new forms/variants of bacteriocins and, consequently, their antimicrobial and apoptogenic effects on various cell lines. For example, Shastry and colleagues discovered a new variant of the class 2 bacteriocin enterocin (enterocin EF35) and its anticancer characteristics [182]. Specifically, a putative tertiary structure of enterocin EF35 was inferred using sequence analysis. After confirming its novelty and structure, the bacteriocin was tested in silico against two proteins of human origin: topoisomerase 1 (TOP1) and phosphoinositide 3-kinase (PI3K). The study results were promising because enterocin EF35 exhibited an inhibitory effect against both TOP1 and PI3K [182].

### 4.3. Urolithins

Urolithins are a class of secondary metabolites produced primarily by the gut microbiota as a result of the processing of polyphenols, ellagitannins, and ellagic acid [183]. There are different classes/types of urolithins along with different biosynthesis pathways that lead to their production [183]. Four known compounds fall under the urolithins umbrella: urolithin A (Uro A), urolithin B (Uro B), urolithin C (Uro C), and urolithin D (Uro D). While all urolithin compounds have been studied for their effects on particular cancer cell lines and how they can be used therapeutically, Uro-A and Uro-B are much more cited and used in the literature than both Uro-C and Uro-D.

#### 4.3.1. Uro-A

Initially, in the case of urolithin A, one study used the CC cancer cell lines HT29 and SW620 to test its effect on different cancer inhibitors and apoptotic-promoting pathways. The findings indicated that in the SW620 cell line, the level of tumor suppressors p-c-RAF and p-PTEN increased, and the levels of phosphorylated AKT and the cellular proliferation regulator mTOR decreased significantly (*p* < 0.001) [184]. This study suggested that Uro-A could play significant roles in both the PI3K/p-AKT/mTOR and p-c-RAF/MEK/p-ERK pathways [184]. Uro-A was also found to inhibit the Bcl-2 pathway, highlighting its apoptotic role [185]. Similarly, another team tackled CC by using the SW480, SW620, HCT 116, and HT-29 cell lines and testing the effects of both Uro-A and Uro-B. The findings were similar to those of El-Wetidy and colleagues in that Uro-A was found to promote the proliferation of anti-CC cells and have anticancer effects on CC cells [186]. The PI3K/Akt/mTOR pathway was also studied by another team that found ties between Uro-A and both tumor suppression (in vivo) and the inhibition of gastric cancer (GC) cell proliferation and migration (in vitro) [187]. Uro-A was also found to be an activator of transcription factor FOXO1, which leads to the expansion of cancer immunosurveillance CD8 T cells [188]. Karim et al. focused on the in vivo aspect by inducing liver injury in Wistar rats using doxorubicin and testing Uro-A in a dose-dependent manner. The results, in line with previous studies, indicate an increase in the antioxidant enzymes SOD and CAT in the liver and an inhibitory effect on the inhibitory cytokines TNF-α, NF-kB, and IL-6 [189]. In addition to the reported antioxidant and anti-inflammatory effects, it was also reported that Uro-A has anticancer and apoptotic effects in that it significantly increased the expression of caspase 3 and cytochrome c oxidase in livers with DOX-induced injuries [189].

#### 4.3.2. Uro-B

Furthermore, while Uro-B is less studied than Uro-A, a substantial amount of the literature still discusses its role as an anticancer agent. One team studied the compound’s effects using male C57BL/6 mice and APC^min/+^ mice as models for CC. The findings describe the prevention of colorectal carcinogenesis through the “remodeling” of the gut microbiota and tumor immune microenvironments, involving HLA-B cells, NK cells, regulatory T cells, and γδ-TCR cells [190]. When the researchers combined Uro-B with the oral chemotherapy drug capecitabine, the mice showed an improvement in colorectal intestinal hematochezia due to the reshaping of their gut microbiota [190]. Another study tackles Uro-B both in vivo (mouse model) and in vitro (HT29 cell line). The findings were, yet again, in line with studies focused on urolithins. In particular, just as with the study conducted by Karim et al. with Uro-A, this study finds that Uro-B also has inhibitory effects on inflammatory cytokines such as IL-6, TNF-α, IFN-γ, IL-4, and IL-1β [191]. Uro-B was also found to suppress the expression of TLR4, IRAK4, TRAF6, IKK-β, NF-κB p65, and HMGB1, all of which are involved in pathways leading to inflammatory responses [191]. The same anti-inflammatory effects were observed in vitro with lipopolysaccharide-induced inflammation in HT29 cells. Overall, there is overwhelming evidence supporting Uro-B as a candidate anticancer therapeutic agent for its role in various cancer-related pathways both in vivo and in vitro [192,193,194,195,196].

#### 4.3.3. Uro-C and Uro-D

Lastly, as mentioned previously, most of the literature available is only concerned with ellagic acid, Uro-A, and Uro-B. It is noteworthy to mention that from one of the few studies conducted on all four members of the urolithin family, Uro-D was cited for having a powerful antioxidant effect in aqueous solutions; the study confirmed this using both in vivo and in silico methods [197]. Another study focused on Uro-C and, like the findings concerning both Uro-A and Uro-B, described its anticancer effects against CC cells. Specifically, the team performed an in vitro experiment by adding Uro-C to the DLD1, HCT116, RKO, and HEK293T CC cell lines in a time- and dose-dependent manner [198]. Uro-C, like Uro-A and Uro-B, was found to block the activation of the PI3K/p-AKT/mTOR pathway [198].

## 5. The Roles of Gut Microbiota Metabolites in the Development of Infectious Diseases 

Trendy academic research suggests triggering secondary gut antimicrobial progressions to influence the benefit of gut microflora and reduce the risk of communicable diseases. Numerous studies have revealed that the misbalancing rate of beneficial and harmful gut microbiota rapidly accelerates pathogenic microbial growth. In addition, multifactorial chemicals ultimately drive gut microbiota stability, for instance, nutrition values, the immune system, gene interactions, and environmental drivers [199,200,201]. A considerable function of the gut microbiota is ultimately as a kind of homeostatic medium for biological activities, such as energy and vitamin production, short fatty acid (SCFS) production, and microbial growth inhabitation [202,203,204,205,206]. Mainly, “dysbiosis” is related to the consequences of changes in the gut microbiota and influences pathogenic bacteria to introduce toxicity. These changes dramatically promote inflammation, disease activity, and immunosuppression [203,207,208]. Commensal or opportunistic bacteria, such as *Bacteroides fragilis* and *Faecalibacterium prausnitzii*, use the unstable gut ecosystem as a factor in spreading their harmful products. In parallel, viral, fungal, and protozoa cells can positively induce their pathogenic attacker into the host cells during environmental changes in the gut microbiota [209,210]. Significantly, previous studies have shown that the gut microbiota plays an essential role in pathogenic progression and the immune response.

### 5.1. Immune Cells

Systemically, the gut microbiota plays a crucial role in protecting and maintaining the human organs to protect and maintain them from endogenous and exogenous disease penetration. The lung is a favored organ by many infectious diseases such as COVID-19, SARS-CoV-2, and influenza viruses. Experimental studies have pointed to viral pathogenic diseases influenced by harmful bacterial products. Lipopolysaccharides have been to increase the potential interest of virus cells in health cells and alveolar cells [206,211,212]. This disequilibrium alerts the immune system to increase T-cells and antibodies to reduce the spread of harmful chemicals; however, virus cells tolerate the protection line and invade cells to impair the function of healthy cells. The gut microbiota mainly initiates *Staphylococcus epidermidis* as a crucial component of mucus to regulate the protection of the host organs. *Staphylococcus epidermidis* secures the lung cells from injuries and inflammation in the respiratory tract. It eliminates the progression and spread of pathogenicity of foreign invaders such as viruses. The results of Kim’s experiment, which uses the influenza A virus (IAV) infection mouse model, have demonstrated that the concentration of *S. epidermidis* cells improves the immunity sensitivity of virus development [213].

Furthermore, pathogenicity mechanisms are controlled by the immune system, genetics, age, and environmental factors [214,215,216]. Cambell has found that harmful bacteria produce chemical substances that activate the role of Th17 cells and regulate the immune response to foreign metabolites [217]. Th17 immunity cells, myofibroblasts, and epithelial intestinal cells simultaneously produce cytokine bodies that rebuild the damaged tissue, protect healthy cells, and enhance metabolite function in foreign cells such as virus, fungal, and protozoa cells [203,204,205]. It is notable that primary bile acids (PBAs) and secondary bile acids (SBAs) interchangeably facilitate each other for intestinal stability; however, studies have highlighted that the excessive stimulation of SBA elements promotes infectious microbial proliferation. Fungal diseases such as *Candida albicans* and coronavirus increase their infectivity level during the high level of SBAs and coronavirus species as well [109,218]. In addition, short-chain fatty acids (SCFAs) notably play a vital function in the gut microbiota to support the other organic cells. The existence of the butyrate protein can maintain liver metabolism; on the contrary, more stimulation of SCFAs could accelerate injuries to the liver, for example, hepatocyte cell death and hepatic B and C viruses [191,219,220,221]. In addition, it was reported that high-density lipoprotein (HDL) neutralizes the toxic products of infectious progression in the liver. Although the liver excretes the systematic HDLs, the gut microbiota produces apolipoprotein A1 (apoA1), which regulates liver inflammation [191]. The most highlighted finding of Jieun and Ekihiro’s study is that the liver is where the gut microbiota adjusts HDL and the accompanying lipopolysaccharide (LPS). The lipopolysaccharide (LPS) drives liver cells into the healthy stage and diminishes the risk of liver inflammation and injuries [222].

### 5.2. Inflammatory Bowel Diseases (IBD) and Infectious Diseases

Generally, inflammatory bowel diseases (IBDs) are caused by complex changes in gut microbial equilibrium levels, which influence commensal bacterial and viral cells to grow. IBD is well-known to regulate intestinal mucus for harmful bacterial inhabitants, reduce immune cell function, and activate pathogenic and inflammation cell interactions [200,223]. The relationship between opportunistic bacteria such as *Escherichia coli* (AIEC) and IBD has been highlighted in the literature [203,224,225,226]. 

## 6. The Potential of Gut Microbiota Metabolites as Infectious Disease Therapeutics

An enormous number of microbes is present in the human intestine, constituting a complex ecological environment that impacts normal physiology and vulnerability to disease immune responses through interactions with the host and metabolic activities [227]. Gut microbiota metabolites can stimulate different modulators of the immune response, such as regulatory T cells, gut-associated immune tissue, lymphoid tissues, and the mucosal barrier [228].

The widely explored metabolite classes of the gut microbiota with physiological and therapeutic effects on humans include organic acids, branched-chain fatty acids, short-chain fatty acids, lipids, branched-chain and aromatic amino acids, vitamins, bile acids, neurotransmitters [229], and tryptophan metabolites [230].

Some bacteria, such as Bifidobacterium BB-12, secrete hydrogen peroxide, which is toxic and harmful to surrounding pathogenic bacteria. This favors effective colonization of the area where the bacteria are present in the gut [231].

### 6.1. Bile Acid Metabolites

Bile salt substrates demonstrated a suppressive influence on the replication of the hepatitis B virus (HBV). They have the ability to reduce the binding of viral pre-S1 to the sodium taurocholate transporting polypeptide, which is a cellular receptor for human hepatic B and D viruses and is required for entry into hepatic cells [232], as shown in Table 3. The taurine conjugate form of ursodeoxycholic acid, a bile acid metabolite, is also a potent inhibitor for hepatitis infection (Table 3). Recent clinical observations have suggested that prolonged treatment with bile salts, particularly ursodeoxycholic acid, may contribute to the enhancement of liver function in patients diagnosed with hepatitis B (Table 3) [233]. Tryptophan-derived bacterial metabolites such as indole, tyramine, indole-3-acetate, and skatole have been demonstrated to be AhR agonists. This receptor modulates inflammatory responses, hence their anti-inflammatory properties in the liver [234].

### 6.2. Short-Chain Fatty Acids

Butyrate is one of the critical components of SCFAs, and it has several biological effects. Butyrate can induce the programmed death of hepatoma cells and prevent cell proliferation [156]. Gut microbiota metabolites can induce and facilitate host immune interaction with viral agents. Gut microbiota-derived butyric acids promote the preservation of CD8+ T cells and improve the memory potential of activated CD8+ T cells by uncoupling the tricarboxylic acid cycle from glycolytic input, facilitating a more effective immunological response to antigen re-encounter [244]. 

They can enhance the responsiveness of the immune system to infectious agents. SCFA treatment has been associated with reduced ACE 2 expression, decreased viral loads, and enhanced adaptive immunity [245]. The mechanisms of action of SCFAs at different levels include histone deacetylase inhibition, G protein-coupled receptor interactions, reductions in the expression of several cytokines, increased Treg cell numbers and function, enhancement of gut integrity, and decreased serum lipopolysaccharide levels [151]. It was discovered that SCFAs appear to be a natural ligand for free fatty acid receptors 2 and 3, which are found on a wide range of cell types, including enteroendocrine and immune cells. The influence of SCFAs on cellular function is evidently multifaceted, as they can directly or indirectly modulate the activity of various cell types, including epithelial cells and both innate and adaptive immune cells [246]. Promoting regulatory and effector T cell development, T cell memory, and plasma cell differentiation are some mechanisms through which SCFAs regulate the immune response [247]. SCFAs have been shown to enhance antiviral T cell responses and boost the activation of spike-specific B and T cells, leading to improved antibody neutralization following SARS-CoV-2 infection (Table 3) [245]. Fukuda’s team [235] found that acetate, a short-chain fatty acid produced by commensal bacteria in the distal colon, could offer protection against *E. coli* O157-induced mortality in mice, along with providing various beneficial therapeutic effects on the host, including trophic and anti-inflammatory properties.

Acetone also induces interferon-β (IFN-β) in the lung and confers protection against infectious diseases mediated by G protein-coupled receptors (GPR43) and interferon-α/β receptors (IFNAR) [236]. Commensal bacteria play a crucial role in enhancing the cell-mediated immune response to tuberculosis (Table 3). They reduce inflammation and protect against lung injury by producing secondary metabolites during their interactions with the host microbiome [248].

### 6.3. Indole

Intestinal commensals, such as *Clostridia* species, produce a significant and well-tolerated metabolite known as indole propionic acid through tryptophan metabolism. Research has shown that this compound exhibits anti-tuberculosis properties in mouse models. In mice with acute tuberculosis, indole propionic acid was found to reduce the *Mycobacterium tuberculosis* load in the spleen by sevenfold, suggesting its potential therapeutic benefits in the treatment of tuberculosis (Table 3) [240]. Through the circulation, secondary metabolites of gut microbiota and immune signals travel between the lungs and intestines in the bloodstream [249], which is crucial for gut microbiome metabolites to affect their therapeutic action in the lungs through the lung–gut axis.

## 7. Techniques for Gut Microbiome-Based Therapy in Pathogenic Disease

### 7.1. Fecal Microbiota Transplantation

Fecal microbiota transplantation functions through several essential mechanisms, starting with the introduction of a diverse population of beneficial microbes from a healthy donor into the gastrointestinal tract of a patient exhibiting dysbiosis at both compositional and functional levels. This newly introduced microbiota competes with and replaces pathogenic bacteria, contributing to the restoration of a healthy gut microbiome balance. Fecal microbiota transplantation (FMT) is primarily utilized for the treatment of *Clostridium difficile* infections, which often occur as a result of antibiotic use. However, its applications extend beyond this, demonstrating potential benefits in the management of several other conditions, including colorectal cancer, ulcerative colitis, Crohn’s disease, metabolic syndrome, obesity, and autism. Unlike other organ transplants, FMT provides a distinct safety advantage, as it integrates into the existing microbiota without the risk of rejection by the body [250].

This approach represents the most effective method for restoring intestinal microecology and addressing both intestinal and extraintestinal diseases [251].

### 7.2. Microbiome Engineering

Microbial engineering involves altering the structure of the gut microbiome for therapeutic benefits, an innovation that the food and pharmaceutical industries have recently been exploring [252]. Microbiome engineering is primarily achieved through several methods, including probiotics, prebiotics, antibiotics, bacteriophage administration, and microbiota transplants. Probiotics play a crucial role in influencing the gut microbiome through mechanisms such as competition, antimicrobial production, and immune modulation. Prebiotics selectively enhance the growth of beneficial microorganisms. Although antibiotics can effectively eradicate pathogens, they may lead to a reduction in microbial diversity. Intestinal microbiota transplantation has been shown to help prevent the recurrence of *Clostridium difficile* infections. Additionally, bacteriophages present a promising approach for targeting specific intestinal pathogen strains [253].

Prophylactic and therapeutic strategies for human microbiome engineering also entail the modification of the composition of the microbiome community, adjusting their metabolic activities, facilitating microbe–microbe interactions, and regulating host–microbe relationships. Non-pathogenic *Escherichia coli* has been engineered to detect and combat *Pseudomonas aeruginosa*, a human opportunistic pathogen linked to chronic cystic fibrosis and gastrointestinal colonization. The engineered *E. coli* detects a small molecule from *P. aeruginosa’s* quorum sensing, triggering a self-lysis program that releases a targeted bacteriocin to kill the pathogen. Similar methods have been adapted to respond to Vibrio cholera infections by sensing autoinducer-1 (AI1) from its quorum sensing pathway [254].

Research indicates the potential of microbiome engineering through the use of genetically engineered *Saccharomyces boulardii* or *Saccharomyces cerevisiae* as a probiotic for the production of recombinant proteins in the intestine. This may aid in the treatment of inflammatory bowel disease and the specific treatment of *Clostridium difficile* infection [255].

### 7.3. Microbiome-Based Vaccines

Some probiotics have surface structures that can elicit immune responses. They have been explored as vaccine vectors that offer simplicity and a non-invasive route of administration, typically oral or intranasal, alongside the acceptance and stability of genetic modifications. They also provide a relatively low-cost solution with an emphasis on achieving the highest possible safety standards. Lactic acid bacteria, especially those belonging to the Lactobacillus genus, have demonstrated significant potential as mucosal vectors, effectively eliciting both systemic and mucosal responses, particularly when used in conjunction with adjuvants [256].

### 7.4. Bacteriophages

Bacteriophages are naturally occurring viruses that specifically target bacterial cells. They play a vital role in the colonization of intestinal bacteria and the regulation of bacterial metabolism [257]. Phage therapy leverages bacteriophages by exploiting bacterial cellular mechanisms for reproduction through the transduction process. Bacteriophages comprise nucleic acid (DNA or RNA) enclosed within a protein capsid. They adhere to specific receptors on bacterial surfaces and then transfer their genetic material to complete their lifecycle through one of two pathways: the lytic cycle, which leads to the release of new phage progeny following the lysis of the bacterial cell, or the lysogenic cycle, in which the phage genome is integrated into the bacterial genome [258]. 

Bacteriophages have been explored in the management of bacterial infections during both the prophylactic and therapeutic phases. They are known to possess significant potential in therapeutic applications for several critical bacterial diseases where antibiotics and other treatment options may not be effective [259]. Bacteriophages have been used in the management of bone diseases, upper and lower respiratory infections, skin and soft tissue infections, urinary tract infections, and eye and ear infections [260]. Phage therapy can also be used to eliminate antibiotic-resistant bacteria [261]. It has also been used to target several intracellular bacterial pathogens such as *Chlamydia* spp., *Klebsiella pneumoniae*, *Salmonella enterica*, and *Pseudomonas aeruginosa* [262]. 

## 8. Interaction Between Microbiota and microRNAs

The interplay between microbiota and microRNAs (miRNAs) represents a burgeoning area of research with profound implications for understanding host–microbe communication and its impact on health and disease. Microbiota, the diverse communities of microorganisms residing in various areas of the human body, particularly the gut, can influence miRNA expression profiles in host tissues. Conversely, host-derived miRNAs can regulate microbiota composition and function, establishing a bidirectional communication system. Microbial metabolites, such as short-chain fatty acids (SCFAs), bile acids, and lipopolysaccharides, have been shown to modulate host miRNA expression [263]. These metabolites can activate specific signaling pathways, such as the NF-κB or MAPK pathways, leading to alterations in miRNA transcription. For example, butyrate, an SCFA produced by gut bacteria, has been demonstrated to upregulate miR-375, which plays a role in maintaining intestinal epithelial integrity.

Additionally, bile acids influence miRNA expression by interacting with nuclear receptors such as FXR, thereby modulating lipid metabolism and inflammatory responses. Dysbiosis, or microbial imbalance, can result in aberrant miRNA expression, contributing to inflammatory and metabolic disorders [264]. Furthermore, these microbial signals can have systemic effects, influencing miRNA profiles in distant tissues, including the liver and brain, highlighting the far-reaching impact of microbiota on host physiology.

Host-derived miRNAs secreted into the gut lumen can be taken up by microbial communities [265]. These miRNAs target bacterial genes, modulating microbial growth and virulence. For example, miRNAs such as miR-515-5p and miR-1226-5p selectively influence the abundance of certain bacterial taxa [266]. By affecting bacterial gene expression, miRNAs play a crucial role in maintaining microbial balance and promoting beneficial interactions. This regulatory mechanism highlights the importance of miRNAs in cross-kingdom communication and host–microbiota homeostasis.

Therapeutic strategies targeting this axis are emerging as promising approaches. Probiotics and prebiotics can influence miRNA expression by reshaping microbial communities, as seen with strains of Lactobacillus that promote anti-inflammatory miRNAs. Additionally, fecal microbiota transplantation (FMT) has shown potential in restoring healthy miRNA profiles and microbial balance, particularly in cases of recurrent *Clostridium difficile* infection. FMT has been associated with the modulation of circulating and intestinal miRNA levels, contributing to therapeutic effects in recurrent infections [267]. Advances in miRNA-based therapeutics, such as miRNA mimics or inhibitors, provide tools to target dysregulated pathways directly. These interventions hold promise for addressing complex disorders rooted in the microbiota–miRNA axis, emphasizing the therapeutic potential of this intricate communication network. As research progresses, these therapies could become integral components of personalized medicine, offering targeted solutions for microbiota-related diseases.

## 9. Discussion

The gut microbiota is a highly dynamic ecosystem that significantly influences host physiology, with its metabolites playing critical roles in modulating immune pathways, epigenetic mechanisms, and disease progression. This review synthesizes current findings on the therapeutic potential of gut microbiota metabolites, particularly in gastrointestinal (GI) cancers and infectious diseases. We highlighted how metabolites such as short-chain fatty acids (SCFAs), indoles, polyamines, and secondary bile acids exhibit dual roles in both promoting and suppressing disease. These compounds mediate effects ranging from tumorigenesis and inflammation to tumor suppression and immune modulation, underscoring their importance as therapeutic targets. This review also emphasized the intricate interplay between microbial metabolites and epigenetic regulation, illustrating novel pathways through which they influence disease outcomes and therapeutic efficacy. Despite significant advancements, the field of microbiota metabolite research remains ripe for exploration. There are notable gaps in our understanding of how specific metabolites influence the heterogeneity of patient responses to therapies. In addition, the dual role of certain metabolites, such as secondary bile acids, in disease progression and suppression highlights the need for further elucidation of context-specific mechanisms.

Future studies should focus on characterizing the mechanisms through which microbial metabolites influence therapy responses and disease heterogeneity. Emerging technologies such as high-resolution metabolomics and single-cell sequencing can uncover new metabolite pathways and their impact on host immunity and epigenetics. Recent evidence highlights butyrate’s role as a histone deacetylase (HDAC) inhibitor that can modulate immune function and suppress tumor growth, emphasizing its potential for cancer epigenetic therapy [268]. Additionally, the role of microbial metabolites in enhancing immunotherapy efficacy deserves further attention. For instance, novel research indicates that gut metabolites can modulate immune checkpoint inhibitors by influencing T-cell activation and exhaustion pathways, providing new avenues for personalized cancer treatments [269]. In infectious disease research, understanding how metabolites mediate resistance to pathogens and antimicrobials could lead to innovative strategies to address global challenges such as antimicrobial resistance.

Engineered probiotics and synthetic metabolite analogs represent promising tools for precise therapeutic interventions. Integrating dietary modulation, such as fiber- or polyphenol-rich diets, with these tools could amplify the production of beneficial metabolites, fostering a synergistic approach to treatment. Furthermore, exploring the interplay between the gut microbiota and host genetics may unlock insights into individualized therapies that harness the full therapeutic potential of microbial metabolites. By addressing these research priorities, future work can translate the immense potential of gut microbiota metabolites into actionable strategies for managing cancer, infectious diseases, and antimicrobial resistance.

## 10. Conclusions

The gut microbiota is a complex community that significantly influences human health. Its metabolites play a dual role in cancer development, promoting tumor growth through inflammation and DNA damage while also stimulating apoptosis and enhancing immune responses. Additionally, the gut microbiota can affect the effectiveness of anti-tumor therapies, potentially sensitizing tumor cells to chemotherapy or inducing drug resistance. Changes in gene expression due to microbiota products can contribute to disease development. Furthermore, these metabolites may impact infectious diseases by altering susceptibility and interacting with pathogens. Understanding the relationship between the gut microbiota and health could lead to innovative therapies for cancer, epigenetic disorders, and infectious diseases.

## Figures and Tables

**Table 1:** Role of gut microbiota metabolites in tumorigenesis and tumor treatment. G—genus, F—family, P—phylum.

Secondary Metabolite	Bacterial Phyla	Associated Cancer	Biological Process	References
Hydrogen sulfide	*Desulfovibrio vulgari*	CRC	cytotoxic DNA damage	[40,41,42]
Secondary bile acids	Bacteroidetes (P) Firmicutes (P) Actinobacteria (P)	CRC	DNA damage Inhibit apoptosis Upregulate IL-8	[35,41,57,58]
Lactate	*Streptococcus thermophilus*	CRC	Increase PH value of TME	[41]
*Bacteroides fragilis* toxin	*Enterotoxigenic Bacteroides fragilis*	CRC	Increase pro-inflammatory factors DNA damage	[42,59,60]
Colibactin	*Escherichia coli*	CRC	Mutation DNA damage Chromosomal instability	[43,60,61,62]
Cytolethal distending toxin	Proteobacteria (P) *Campylobacter jejuni*	CRC, HCC	DNA double-strand break	[43,49]
Adhesin A toxin	*Fusobacterium nucleatum*	CRC	-	[44]
Butyrate	*Eubacterium rectale Roseburia hominis* *Akkermansia muciniphila* Bifidobacterium (G) Ruminococcus Faecalibacterium Coprococcus (G) Oscillibacter (G) Clostridium IV (G) Verrucomicrobia (G)	HCC	promote host cell differentiation, inflammation, apoptosis, and intestinal mucosal integrity upregulate calcium signaling pathways	[45,46,63,64]
	Blautia (G) Lachnospiraceae (G) Eubacterium (G) Bacteroides (G) Ruminococcaceae (G)	CRC	increase oxaliplatin sensitivity	[65]
Lipopolysaccharide	Klebsiella (G) Haemophilus (G) Prevotella (G) Parabacteroids (G) Paraprevotella (G) Mucispirillum (G) Alistipes (G) Oscillibacter (G) Butyricimonas (G) *Porphyromonas gingivalis* *Fusobacterium nucleatum*	HCC, EC, PC	Increase pro-inflammatory cytokines disrupt the epithelial tight junctions, activate the NF-kB pathway	[45,46,47,56,66,67,68,69,70,71]
cytotoxic-associated gene A	*Helicobacter pylori*	GC	Mutation, Stimulate IL production	[72,73]
Nisin	*Lactobacillus lactis*	HCC	Increase caspase-3 levels (apoptosis)	[74]
		HNSCC		[75]
Pediocin	*Pediococcus acidilactici* K2a2-3	Lung cancer, HCA, CC	Inhibit cell progression	[76,77]

**Table 2:** Gut microbiota and their effect on healthy individuals and colorectal cancer (CRC) patients.

Items	Healthy	CRC
Gut microbiota	Butyrate producers Lactate producers *Lactobacillus*	Sulfur reducers Secondary bile acids producers *E. coli* *Fusobacterium nucleatum*
Gut microbiota impact	Decreased gut inflammation Improved immune system Increased antioxidant production Increased short-chain fatty acid production	Increased toxin production Lipopolysaccharide production Increased cell proliferation DNA damage Increased gut inflammation
References	[42,43]	[36,37,38,39,40,43,44]

**Table 3:** Role of gut microbiota metabolites in the treatment of infectious diseases.

Gut Metabolites	Microbes/Bacterial Phyla Producing Them	Disease(s) They Affect	Biological Process	References
Acetate	Firmicute, Bacteroidetes, Actinobacteria	*Escherichia coli* O157	Trophic and anti-inflammatory properties	[235]
Acetate	Firmicute, Bacteroidetes, Actinobacteria	Respiratory syncytial virus	GPR43-type 1 interferon response	[236]
Acetone	Lactobacillus, Enterococcus, Streptococcus, and Enterococcaceae	Tuberculosis	Induce G protein-coupled receptors (GPR43) and interferon-α/β receptors (IFNAR)	[236]
Butyrate	*Escherichia coli* MG1655 and *E. coli* (Nissle 1917; EcN)	Hypervirulent *Klebsiella pneumoniae* and *Salmonella enterica Typhimurium*, opportunistic nosocomial pathogen *E. cloacae*, and antibiotic-resistant strains of *K. pneumoniae*, *E. coli*, and *Proteus mirabilis*	Activation of host signaling peroxisome pathways	[237,238]
Deoxycholate	*Clostridium cluster XIV*	*Clostridium difficile* infection (CDI)	Inhibit germination, growth, and toxin activity	[239]
Indole	Bacteroides, Firmicutes, and Verrucomicrobiota	Hepatitis, *Cryptosporidium parvum* infection, and Tuberculosis	AhR agonists decrease membrane potential within the parasite’s mitosome	[234,240,241]
Tryptamine	Ruminococcus, Firmicutes, Bacteroidetes, and Actinobacteria	COVID-19	5-hydroxytryptamine receptor [5-HTR] agonist reducing cellular entry	[242]
Ursodeoxycholic acid	Bacteroides	Hepatitis B virus (HBV) disease	Prevent cellular entry and Evasion through farnesoid-X Receptor	[232,233,243]

## Data Availability

No datasets were generated or analyzed during this study.

## Abbreviations

ACADS	Acyl-CoA Dehydrogenase Short Chain
ACE	Angiotensin I Converting Enzyme
AhR	Aryl Hydrocarbon Receptor
AIEC	Adherent-Invasive E. coli
Bas	Bile Acids
CagA	Cytotoxic-Associated Gene A
CAT	Catalase
SOD	Superoxide Dismutase
CC	Cervical Carcinoma
CDD	Cytidine Deaminase
CHAC1	ChaC Glutathione Specific Gamma-Glutamylcyclotransferase 1
CLDs	Chronic Liver Diseases
CpG-ODN	CpG-Oligodeoxynucleotide
CRAMP	Cathelicidin-Related Antimicrobial Peptide
CRC	Colorectal Cancer
CRP	C-Reactive Protein
CSF	Cerebrospinal Fluid
CTLA-4	Cytotoxic T-Lymphocyte-Associated Protein-4
CTX	Cyclophosphamide
DCA	Deoxycholic Acid
EAC	Esophageal Adenocarcinoma
EC	Esophageal Cancer
ECH	Echinacoside
EMT	Epithelial-to-Mesenchymal Transition
ERK	Extracellular Signal-Regulated Kinase
ERK1	Extracellular Signal-Regulated Kinase 1
ESCC	Esophageal Squamous Cell Carcinoma
FMT	Fecal Microbiota Transplantation
FOXO1	Forkhead Box O1
5-FU	5-Fluorouracil
FXR	Farnesoid X Receptor
GC	Gastric Cancer
GI	Gastrointestinal
GPR43	G Protein-Coupled Receptor 43
HBV	Hepatitis B Virus
HCA	Human Colon Adenocarcinoma
HCC	Hepatocellular Carcinoma
HDAC	Histone Deacetylase
HDACi	Histone Deacetylase Inhibitor
HDL	High-Density Lipoprotein
HEK293T	Human Embryonic Kidney 293T Cells
HLA-B	Human Leukocyte Antigen B
HMGB1	High Mobility Group Box 1
HNSCC	Head and Neck Squamous Cell Carcinoma
HSD	High Salt Diet
HUVEC	Human Umbilical Vein Endothelial Cells
IAA	Indole-3-Acetic Acid
IAV	Influenza A Virus
IBD	Inflammatory Bowel Disease
ICIs	Immune Checkpoint Inhibitors
IFN-γ	Interferon-Gamma
IFN-β	Interferon-Beta
IFNAR	Interferon-Alpha/Beta Receptors
IKK	IκB Kinase
IL-1β	Interleukin-1 Beta
IL-10	Interleukin-10
IL-12	Interleukin-12
IL-2	Interleukin-2
IL-4	Interleukin-4
IL-6	Interleukin-6
IL-8	Interleukin-8
IRAK4	Interleukin-1 Receptor-Associated Kinase 4
KRAS	Kirsten Rat Sarcoma Viral Oncogene Homolog
LAB	Lactic Acid Bacteria
LC3-II	Microtubule-Associated Protein 1A/1B-Light Chain 3
LCA	Lithocholic Acid
LPS	Lipopolysaccharide
MAPK	Mitogen-Activated Protein Kinase
MEK	MAPK/ERK Kinase
mTOR	Mechanistic Target of Rapamycin
MTT	Methyl Thiazolyl Tetrazolium (Assay)
MyD88	Myeloid Differentiation Primary Response 88
NaAc	Sodium Acetate
NaBu	Sodium Butyrate
NaPr	Sodium Propionate
NF	Nuclear Factor
NF-kB	Nuclear Factor Kappa B
NK	Natural Killer (Cells)
ODN	Oligodeoxynucleotide
PBAs	Primary Bile Acids
PC	Pancreatic Cancer
PD-1	Programmed Cell Death Protein-1 Receptor
PD-L1	Programmed Cell Death Protein-Ligand 1
PDAC	Pancreatic Ductal Adenocarcinoma
PI3K	Phosphoinositide 3-Kinase
PTEN	Phosphatase and Tensin Homolog
RAF	Rapidly Accelerated Fibrosarcoma Kinase
ROS	Reactive Oxygen Species
SBA	Secondary Bile Acids
SCFAs	Short-Chain Fatty Acids
TCR	T Cell Receptor
TJs	Tight Junctions
TLR	Toll-Like Receptor
TME	Tumor Microenvironment
TNF	Tumor Necrosis Factor
TOP1	Topoisomerase 1
TRAF6	TNF Receptor-Associated Factor 6
TWIST1	Twist Family BHLH Transcription Factor 1
VacA	Vacuolating Cytotoxin A
AI1	Autoinducer-1
